# An Assessment of the Contrast Sensitivity in Patients with Ametropic and Anisometropic Amblyopia in Achieving the Corrected Visual Acuity of 1.0

**DOI:** 10.1038/srep42043

**Published:** 2017-02-07

**Authors:** Guipan Wang, Congling Zhao, Qiang Ding, Ping Wang

**Affiliations:** 1 Department of Ophthalmology, Tongji Hospital, Tongji Medical College, Huazhong University of Science and Technology, Wuhan, Hubei Province, China; 2 Aerospace 731 Hospital, Beijing, China; 3 Wuhan Aier Eye Hospital, Wuhan, Hubei Province, China; 4 Department of Gastroenterology, Tongji Hospital, Tongji Medical College, Huazhong University of Science and Technology, Wuhan, Hubei Province, China

## Abstract

Both visual acuity (VA) and contrast sensitivity (CS) are important parameters for measuring visual function. In this research, we investigated the CS of patients with ametropic or anisometropic amblyopia, whose corrected visual acuity (CVA) recovered to 1.0. Fifty-five cases with amblyopia and 22 control cases with a normal visual acuity of 1.0 were enrolled. The CS of the patients whose ametropic amblyopia had recovered to a CVA of 1.0 at 18 cpd spatial frequency was still lower than that of the normal control group under both photopic and scotopic conditions (P = 0.001, 0.025), but there were no significant differences at low- and middle-spatial frequencies. The CS of amblyopic eyes of the patients with anisometropic amblyopia was lower than that of the normal control group at the 18 cpd spatial frequency under photopic conditions (P = 0.005), and at the 6 cpd, 12 cpd, and 18 cpd spatial frequencies under scotopic conditions (P = 0.008, <0.001, 0.004, respectively). The CS between the amblyopic eyes and the sound eyes of patients with anisometropic amblyopia presented significant differences at the 6 cpd, 12 cpd, and 18 cpd spatial frequencies under scotopic conditions (P = 0.025, 0.045, 0.019, respectively). We suggest that amblyopia treatment should involve not only the correction of VA but also the improvement of CS.

Amblyopia, which is the most common visual disorder among children, is a neurological disorder of binocular vision resulting from a disrupted visual experience during early visual development^1^. It affects visual acuity (VA), contrast sensitivity (CS), visual field, and stereoacuity^2^. VA and CS are two different but important parameters that are used to measure visual function. VA describes visual function under high contrast conditions^3^. CS testing, which characterizes the visual function under different spatial frequencies and different luminance, is another powerful technique to quantify the capability of the visual system^4^. However, CS is often neglected in clinical research. Compared with VA, CS can better describe the visual function in some cases^5^. Our research investigated the changes in CS among patients with ametropic or anisometropic amblyopia in achieving the corrected visual acuity (CVA) of 1.0.

## Material and Methods

### Participants

Patients with lower visual acuity than their normal peers were selected. All patients who cooperated with us in this study had no strabismus, no non-centric fixation, no keratopathy, and no cataracts or other diseases in their eyes. Fifty-seven cases (male 34, female 23; mean, 8.51 years; SD, 1.894 years) with ametropic and anisometropic amblyopia whose CVA had recovered to 1.0 after amblyopia therapy and 22 cases (male 17, female 5; mean, 8.23 years; SD, 1.541 years) with normal visual acuity of 1.0 were enrolled in this study between May 2011 and November 2012. Among the 57 cases with ametropic or anisometropic amblyopia, there were 34 cases (the spherical equivalent is 4.24 ± 2.130DS) with middle or high hyperopia of both eyes and 23 cases (the spherical equivalent of the amblyopic eye is 4.32 ± 2.175 DS, and the spherical equivalent of the non-amblyopic eye is 2.00 ± 1.663 DS) with anisometropia.

All patients were divided into three groups: the ametropic amblyopic group (MA), anisometropic amblyopic group (NA), and the normal control group (NC). The difference in spherical power and the astigmatic power between the two eyes was at least 1.5D and 1.0D, respectively, in the anisometropic amblyopic group.

### Ethical approval and informed consent

Research protocol was approved by the Ethics Committee of Tongji Hospital, Tongji Medical College, Huazhong University of Science and Technology. The informed consents about the follow-up examination were obtained from all the patients. All experiments were performed in accordance with the Declaration of Helsinki.

### Measurements of visual acuity

The measurements of visual acuity employed the international standard vision acuity chart (E chart, Shengde Company, China), the background luminance of which was 300 cd/m^2^ by a LCD monitor. The 1.0 lines of the chart were at the same height as the eyes. The chart was 5 meters away from the subject.

### Measurements of contrast sensitivity

The refractive error of all patients with amblyopia was corrected by wearing glasses. The sinusoidal gratings were used as the contrast sensitivity testing standards to conduct the Functional Acuity Contrast Test (OPTEC^®^ 6500 visual function tester (Stereo Company, U.S.)). The brightness values were 85 cd/m^2^ (light) and 3 cd/m^2^ (dark), respectively. The spatial frequencies of the sinusoidal grating were 1.5 cpd, 3.0 cpd, 6.0 cpd, 12.0 cpd, and 18.0 cpd. The right and left eyes were tested, separately. The test was carried out in the following steps: The remote switch was turned on to simulate the test distance of 6 m. Then, the Day button was pressed to test the bright mode using the 5 # to 9 # test pictures. Finally, the Night button was pressed for the dark mode test. If the answer was correct, the next test picture was presented; if not, the last correct answer was recorded^6^.

### Statistical analyses

The data were analysed using a paired t-test by the Statistical Package for Social Sciences (SPSS) software (Version 17.0). P-values less than 0.05 were considered statistically significant.

## Results

**Figure 1:**
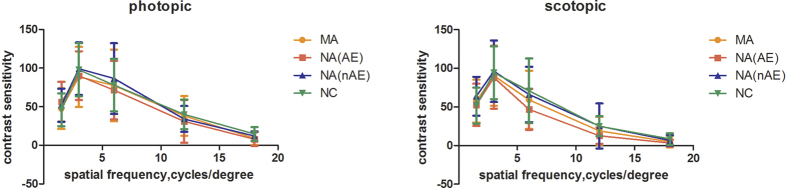
hows the contrast sensitivity of the three groups under the photopic conditions and the scotopic conditions, respectively. Error bars show 95%CIs. P-value of <0.05 was considered statistically significant.

Table 1 and Fig. 1 show the contrast sensitivity of the three groups under photopic and scotopic conditions.

### The differences between the ametropic amblyopic group and the normal control group

Table 2 shows that the two groups exhibited significant differences at 18 cpd under both the photopic and the scotopic conditions (P = 0.001, 0.025).

### The differences between the amblyopic eyes (AE) of the anisometropic amblyopic group and the normal control group

Table 3 shows significant differences at 18 cpd under the photopic conditions (P = 0.005) and 6 cpd, 12 cpd,and 18 cpd under the scotopic conditions (P = 0.008, <0.001, 0.004).

### The differences between the non-amblyopic eyes (nAE) of the anisometropic amblyopic group and the normal control group

Table 4 shows no differences between the groups under both photopic and scotopic conditions at all spatial frequencies.

### The differences between the non-amblyopic eyes and the corresponding amblyopic eyes in the anisometropic amblyopic group

Table 5 shows no differences between the non-amblyopic eyes and the corresponding amblyopic eyes in the anisometropic amblyopic group under photopic conditions. Differences were shown at 6 cpd, 12 cpd, and 18 cpd under scotopic conditions (P = 0.025, 0.045, 0.019).

## Discussion

CS is an important parameter in detecting visual dysfunction. There are two pathways from the retina to cortex: the parvocellular and the magnocellular pathways^7^. Both of them could transform and distribute information to the visual cortex (V1)^8^. The parvocellular system is selectively sensitive to middle to high spatial frequencies (low temporal frequencies); however, the magnocellular system is sensitive to a very broad temporal frequency range (low spatial frequencies)^9^. The contrast-sensitivity function (CSF) is controlled by the spatiotemporal characteristics of the visual pathway. The magnocellular system forms the basis of the achromatic CSF and dominates close-to-threshold detection. When the magnocellular system saturates, the parvocellular system dominates the higher contrast detection^10^.

Amblyopia is a disease with visual abnormality, such as visual acuity, binocular function, and contrast sensitivity^11^. Amblyopia showed decreased contrast sensitivity under mesopic conditions and at intermediate spatial frequencies^12^. The visual acuity was significantly better correlated with improved CS during amblyopia treatment^13^. Despite the significant correlation between VA and CS, VA cannot predict CS^14^. VA is a measure of the resolution under high contrast. CS is a measure of the ability to distinguish the visual markers under different levels of contrast. The latter is more adaptively used in daily life. Unlike other studies that focused on the lower visual acuity of patients with ametropic amblyopia or anisometropic amblyopia, our study focused on the contrast sensitivity of those whose visual acuity had recovered to 1.0.

Our study demonstrated that different types of amblyopia still had contrast sensitivity dysfunction at different spatial frequencies, even when the CVA had recovered to 1.0. The CS dysfunction of the ametropic amblyopia emerged at a high spatial frequency. The CS dysfunction of the anisometropic amblyopia was observed at middle and high spatial frequencies, particularly under scotopic conditions. This result indicated that the recovery of the visual function was different from the low spatial frequency to the high spatial frequency during amblyopia treatment, and the parvocellular system remained uncured, even when the CVA recovered to 1.0.

Compared with photopic visual function mediated by the cone cells, mesopic visual function is more complex because it requires that the rod cells and the cone cells interact with each other properly^15^. This might be why the CS under scotopic conditions is more difficult to recover.

Although the visual acuity of patients with amblyopia had recovered to 1.0, their contrast sensitivity did not return to the normal range simultaneously. In other words, these patients had lower resolution capacities than did those with normal contrast sensitivity, especially under a dark environment. Therefore, we suggest that researchers focus on the improvement of visual acuity and the development of contrast sensitivity during the amblyopia treatment. Perhaps future studies should be directed toward evaluating the impact of reduced contrast sensitivity on the quality of life of patients with a history of amblyopia treatment in whom visual acuity was successfully restored to 1.0. Our findings have enriched the body of knowledge about amblyopia treatment in clinical work.

## Additional Information

**How to cite this article:** Wang, G. *et al*. An Assessment of the Contrast Sensitivity in Patients with Ametropic and Anisometropic Amblyopia in Achieving the Corrected Visual Acuity of 1.0. *Sci. Rep.* **7**, 42043; doi: 10.1038/srep42043 (2017).

**Publisher's note:** Springer Nature remains neutral with regard to jurisdictional claims in published maps and institutional affiliations.

## Figures and Tables

**Table 1:** The contrast sensitivity of the three groups.

	Ap	Bp	Cp	Dp	Ep	As	Bs	Cs	Ds	Es
MA	47.49 ± 26.327	88.82 ± 38.902	77.66 ± 46.515	38.13 ± 25.747	9.04 ± 8.317	57.56 ± 27.762	90.32 ± 38.805	59.1 ± 37.617	18.89 ± 18.181	5.08 ± 7.349
NA (AE)	56.48 ± 25.694	90.30 ± 31.504	71.35 ± 38.006	30.87 ± 27.634	7.91 ± 9.030	52.77 ± 27.306	87.95 ± 40.259	46.73 ± 26.460	12.82 ± 10.813	3.27 ± 4.474
NA (nAE)	52.00 ± 21.494	99.22 ± 34.005	86.52 ± 45.696	34.13 ± 16.739	12.00 ± 6.303	63.86 ± 25.068	96.23 ± 39.762	66.09 ± 35.773	25.32 ± 29.226	6.55 ± 6.631
NC	45.91 ± 21.353	97.91 ± 34.381	78.02 ± 34.082	40.14 ± 19.251	14.66 ± 8.923	51.74 ± 23.271	94.29 ± 34.147	70.97 ± 41.799	25.13 ± 12.916	8.53 ± 7.381

Ap, Bp, Cp, Dp, and Ep represent photopic environment space frequencies of 1.5, 3, 6, 12, 18 cpd, respectively. As, Bs, Cs, Ds, and Es represent scotopic environment space frequencies 1.5, 3, 6, 12, 18 cpd, respectively. MA: ametropic amblyopic group; NA: anisometropic amblyopic group; NC: normal control group. AE: the amblyopic eye; nAE: the non-amblyopic eye.

**Table 2:** The differences between the ametropic amblyopic group (MA) and the normal control group (NC).

	Ap	Bp	Cp	Dp	Ep	As	Bs	Cs	Ds	Es
t	0.332	−1.262	−0.047	−0.884	−3.391	1.128	−0.519	−1.469	−1.849	−2.272
P	0.74	0.209	0.962	0.379	0.001	0.262	0.605	0.145	0.067	0.025

**Table 3:** The differences between the amblyopic eyes (AE) of the anisometropic amblyopic group (NA) and the normal control group (NC).

	Ap	Bp	Cp	Dp	Ep	As	Bs	Cs	Ds	Es
t	1.793	−0.884	−0.732	−1.605	−2.926	0.156	−0.648	−2.749	−3.769	−3.026
P	0.078	0.38	0.467	0.113	0.005	0.877	0.519	0.008	0.000	0.004

**Table 4:** The differences between the non-amblyopic eyes (nAE) of the anisometropic amblyopic group (NA) and the normal control group (NC).

	Ap	Bp	Cp	Dp	Ep	As	Bs	Cs	Ds	Es
t	1.106	1.148	0.785	−1.266	−1.548	1.891	0.199	−0.459	0.028	−1.039
P	0.273	0.882	0.438	0.21	0.127	0.064	0.843	0.648	0.978	0.303

**Table 5:** The differences between the non-amblyopic eyes and the corresponding amblyopic eyes in the anisometropic amblyopic group (NA).

	Ap	Bp	Cp	Dp	Ep	As	Bs	Cs	Ds	Es
t	−0.936	1.066	1.635	0.515	1.737	1.636	1.071	2.41	2.128	2.541
P	0.359	0.298	0.116	0.612	0.096	0.117	0.296	0.025	0.045	0.019
